# Application of Laser and Cryogenic Surface Treatment for the Evolution of Surface Morphology in Additively Manufactured Ti-6Al-4V Alloy Samples

**DOI:** 10.3390/ma18235315

**Published:** 2025-11-25

**Authors:** Dorota Laskowska, Monika Szada-Borzyszkowska, Błażej Bałasz, Wiesław Szada-Borzyszkowski, Izabela Bukała

**Affiliations:** 1 Faculty of Mechanical Engineering and Energy, Koszalin University of Technology, Śniadeckich 2, 75-620 Koszalin, Poland; blazej.balasz@tu.koszalin.pl; 2 Branch of the KUT in Szczecinek, Koszalin University of Technology, 78-400 Szczecinek, Poland; wieslaw.szada-borzyszkowski@tu.koszalin.pl; 3 Emtech Iph Group, Krzywa 3, 59-100 Polkowice, Poland; i.bukala@emtechiph.pl

**Keywords:** additive manufacturing, laser powder bed fusion, Ti-based alloy, laser treatment, dry ice

## Abstract

This study investigates the effects of laser and cryogenic (dry ice) surface treatments on enhancing surface characteristics of Ti-6Al-4V titanium alloy components produced using the Selective Laser Melting (SLM) technique. Components produced via additive manufacturing often exhibit increased surface irregularities and residual unmelted powder, which can deteriorate their mechanical strength and resistance to corrosion. In this study, SLM samples manufactured with two laser powers (176 W and 220 W) were subjected to laser cleaning and dry ice blasting under various process parameters. Surface topography and morphology analyses were performed. The obtained results showed that both methods improved surface uniformity and removed contaminants. Dry ice treatment effectively removed loose powder particles and impurities without causing structural changes—the best results were obtained at a pressure of 10 bar. Laser treatment, depending on the focal length, produced varying degrees of surface remelting—from gentle smoothing (500 mm) to intensive thermal effects and microcracks (250 mm). The research confirmed that cryogenic cleaning is an environmentally friendly and safe post-processing method, while laser cleaning enables deeper surface structure modification, requiring further optimization.

## 1. Introduction

In recent years, additive manufacturing methods—such as Laser Powder Bed Fusion (LPBF), often referred to as Selective Laser Melting (SLM) [1]—have attracted growing interest due to their ability to fabricate parts with intricate geometries [2] and customized mechanical characteristics [3]. However, components produced by these techniques typically exhibit considerable surface roughness [4], which can negatively influence their fatigue life and functional performance [5].

A range of post-processing techniques is applied to enhance the surface quality of components made using additive manufacturing. These include methods such as chemical polishing [6], laser-induced cavitation [7], laser-based surface smoothing [8], and abrasive processing in fluidized beds [9].

Chemical polishing is effective in lowering surface roughness but involves the use of hazardous substances like hydrofluoric acid [6]. In contrast, physical methods—such as laser and cavitation polishing—allow for surface refinement without relying on chemical reagents [7,8]. Laser-based techniques, utilizing either pulsed or continuous laser sources, can significantly smooth surfaces and improve resistance to wear and corrosion, all without mechanical or chemical intervention [8]. Additionally, abrasive fluidized bed machining generates compressive stresses in the surface layer, which contributes to enhanced fatigue strength in additively manufactured parts [9].

The surface properties of Ti-6Al-4V alloy fabricated by the L-PBF method require finishing treatment due to its high roughness and the presence of surface imperfections. The application of techniques such as abrasive blasting, mechanical polishing, or electrochemical polishing enables substantial improvement in surface smoothness and the removal of oxide layers. Among these methods, electropolishing demonstrates the highest efficiency in reducing surface roughness (Ra < 1 μm) and eliminating oxidized layers, which is of critical importance for biomedical applications [10].

The study by Okuniewski et al. [11] explored how shot peening and electropolishing affect the mechanical and corrosion behavior of Ti-6Al-4V alloys manufactured both conventionally and through additive techniques. Shot peening improves fatigue and corrosion performance by inducing compressive residual stresses and altering surface texture, although its effectiveness is highly sensitive to processing conditions and the material’s properties. On the other hand, electropolishing lowers surface roughness and promotes the formation of a stable passive film, which is particularly important in biomedical applications [11].

Li et al. [12] investigated the application of laser polishing to minimize surface roughness, reduce cracking, and eliminate porosity in additively manufactured Ti-6Al-4V components. They observed that performing heat treatment after polishing enhanced material density and helped relieve residual stresses. The findings emphasized the need for careful adjustment of process parameters—such as laser power, scan rate, and environmental conditions—to avoid microcrack formation and unfavorable phase changes [12].

Lizzul et al. [13] evaluated the impact of ball-end milling as a finishing method for Ti-6Al-4V parts produced by LPBF. Their findings indicated that the specific microstructural features of additively manufactured material facilitate localized cutting, which contributes to improved surface smoothness compared to traditionally manufactured samples.

It is important to emphasize that different post-processing techniques can significantly affect both the microstructure and mechanical behavior of complex lattice structures produced additively, such as those with Voronoi-type architectures. Adequate surface smoothing improves structural uniformity and mitigates stress concentrations, thereby enhancing the material’s performance under mechanical loading [14]. Recent studies [9,15] highlight that post-processing methods, including laser polishing and abrasive fluidized bed treatment, directly affect porosity, fatigue performance, and corrosion resistance of additively manufactured (AM) components. Maleki et al. [16] indicate that design for 3D printing should inherently consider surface quality requirements and subsequent post-processing from the earliest stages. Furthermore, research shows that managing residual stresses and controlling phase transformations during post-processing heat treatment is essential, as these aspects have a significant impact on the mechanical performance of the final additively manufactured components [17,18,19].

Advanced post-processing methods, including plasma electrochemical polishing (PEP), femtosecond laser processing, and electron beam treatment, are yielding promising results in the surface treatment of additively manufactured Ti-6Al-4V alloy [20,21]. Studies highlight their potential to improve surface roughness, enhance microstructural uniformity, and reduce residual stresses, which is critical for biomedical and aerospace applications.

Recently, dry ice is increasingly recognized as an eco-friendly and safe alternative for cleaning and surface finishing [22,23,24]. It leaves no secondary contaminants, is non-conductive, and sublimates rapidly, making it particularly suitable for biomedical and electronics applications [25]. This approach effectively removes residual powder from additively manufactured components while preserving the underlying microstructure [26]. Studies confirm that dry ice cleaning effectively removes residual powder and contaminants from the surfaces of AM Ti-6Al-4V components, reduces residual stresses, and does not introduce secondary contamination [27,28].

Based on an analysis of various surface finishing and cleaning methods for additively manufactured components—including chemical, physical, and mechanical techniques—an attempt was made to evaluate the effectiveness of selected processes. This work presents a comparative evaluation of laser and cryogenic (dry ice, CO_2_) surface treatments applied to Ti-6Al-4V alloy components produced by Selective Laser Melting (SLM). The aim was to enhance surface quality and improve post-processing efficiency, with a focus on eliminating residual powder and surface contaminants. Special emphasis was placed on reducing surface roughness, a factor that significantly influences the mechanical and functional performance of final parts. The findings contribute to the development of optimized finishing strategies, supporting improved reliability and functionality of additively manufactured components.

## 2. Materials and Methods

### 2.1. Samples Fabrication

The Ti-6Al-4V samples were fabricated using the Selective Laser Melting (SLM) technique on an ORLAS CREATOR^®^ system (O.R. Lasertechnologie GmbH, Dieburg, Germany). The process parameters were chosen based on previous studies [29], which indicated that modifying certain settings can affect the resulting surface roughness of SLM-manufactured parts. Accordingly, two distinct parameter sets were defined, as outlined in Table 1.

The Ti-6Al-4V alloy powder utilized in the fabrication process was sourced from 3D Systems (Rock Hill, SC, USA). Details regarding its chemical composition are listed in Table 2.

Prior to further analysis, the samples underwent mechanical detachment of support structures and were then ultrasonically cleaned in distilled water for 10 min.

### 2.2. Description of Research Sites

#### 2.2.1. Dry Ice Blasting Method

**Figure 1:**
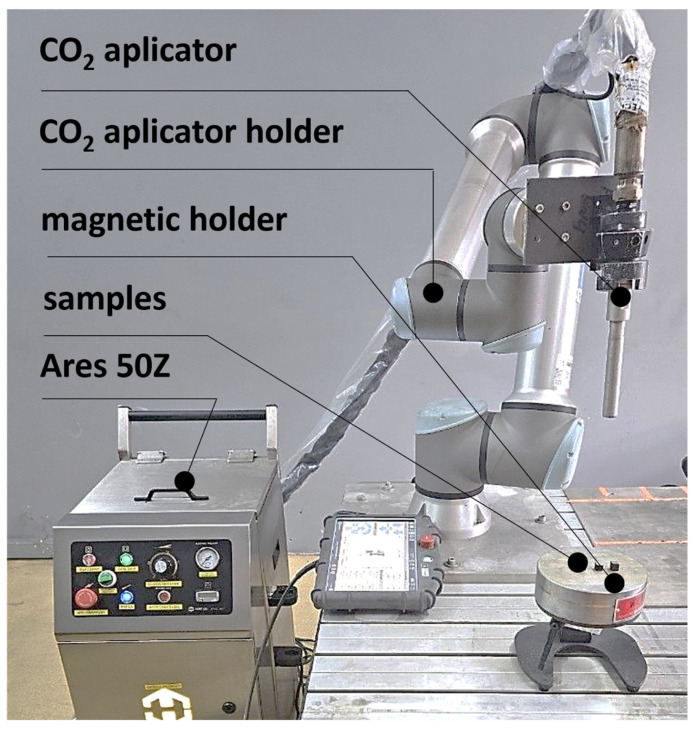
Ares 50Z dry ice blasting device.

The experimental setup for treatment process using dry ice blasting method was equipped with an Ares 50Z device (HORECO_2_, Houxi Industrial Park, Jimei District, Xiamen, China). A view of the device along with the applicator is shown in Figure 1. Table 3 outlines the key operating parameters of the Ares 50Z device.

The surface treatment technique was based on the combined mechanical and thermal action of solid CO_2_ particles, which, upon impact with the sample surface, remove contaminants without altering the material structure. The device allowed for controlled adjustment of process parameters, ensuring high repeatability and operational safety. Depending on the desired effect, CO_2_ particles with an initial maximum diameter of 3 mm were directed to a crushing and feeding system known as a splitter. The splitter fragmented and separated the CO_2_ particles into smaller fractions (0.3–0.5 mm). Before being ejected through the nozzle channel, the crushed particles were uniformly mixed with compressed air and directed toward the treatment surface. The procedure was performed using the settings listed in Table 4.

For dry ice cleaning, pressures of 4, 6, and 10 bar were selected to evaluate the surface structure. This range reflects typical values reported in studies [27,28] and allows for the evaluation of the process’s effectiveness.

#### 2.2.2. Laser Treatment Method

**Figure 2:**
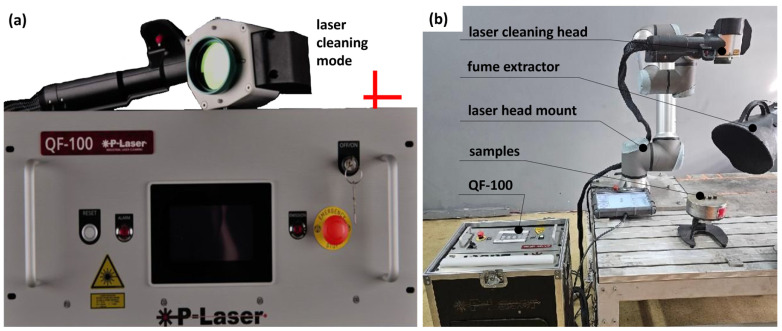
Laser cleaning system QF-100 by P-Laser: (**a**) control unit with cleaning head; (**b**) cleaning setup with laser head, fume extractor, and samples.

In the laser treatment method, a mobile QF-100 laser system (P-LASER ILC, Heusden-Zolder, Belgium) with a power output of 100 W was used. The pulsed QF-100 laser generates short, high-energy pulses. The focused laser beam interacts with the material surface, causing sublimation and vaporization of contaminants without physical contact with the substrate. A view of the device is shown in Figure 2. Table 5 provides the basic technical specifications of the device.

The surface treatment process involved the interaction of a focused laser beam, characterized by a defined shape and laser cleaning mode, with a scanned area diameter of 50 mm. The scanning beam was directed onto the sample surface at a distance determined by the focal length (Table 5). The laser power during each treatment process was set to 100 W.

Focal lengths of 250 mm, 300 mm, and 500 mm were selected to vary the local energy density and evaluate its effect on the range of remelting, ablation, and surface morphology modification. These values reflect a range that allows for both high- and low-intensity interactions, in accordance with studies presented in the literature [8,12,20].

### 2.3. Evaluation of Surface Morphology and Roughness

**Figure 3:**
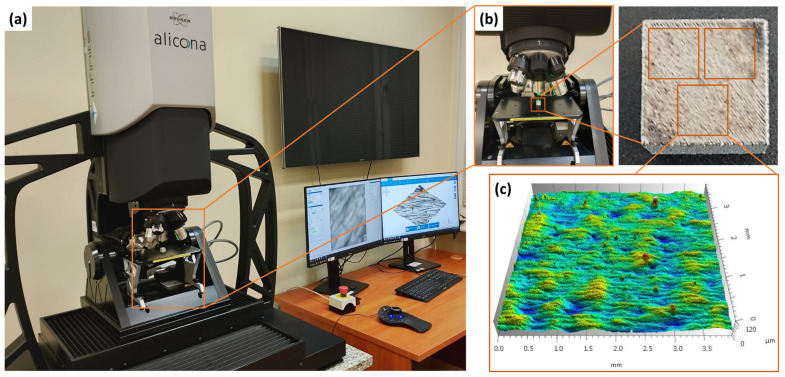
Surface topography measurement station: (**a**) view of the InfiniteFocus G6 3D optical microscope, (**b**) location of measurement areas on the sample surface, (**c**) example view of the analyzed area.

Surface topography measurements before and after laser and dry ice treatment were performed using the InfiniteFocus G6 3D optical microscope (Bruker Alicona, Alicona Imaging GmbH, Graz, Austria) (Figure 3), employing a focus variation technique with a wavelength of 125 nm. To measure the surface, an ×800 WD17 objective was used. The estimated vertical resolution was 0.0301 μm, while the estimated lateral resolution was 3.1313 μm.

In the next stage of the study, surface topography analysis was performed using TalyMap Platinum 7.4 software. Outliers were removed to eliminate measurement noise and obtain a representative surface image.

The surface roughness parameters were defined in accordance with ISO 25178 [30]. A Gaussian filter with a cut-off (nesting index) value of 2.5 mm, as specified in ISO 16610-61 [31] was applied to remove form and waviness components from the data.

This system enabled precise quantification of surface roughness parameters, including the arithmetical mean height (Sa), maximum profile height (Sz), maximum pit depth (Sv), maximum peak height (Sp) and root mean square height (Sq).

For each surface condition analyzed, three separate samples with dimensions of 10 × 10 × 10 mm were prepared. Measurements were conducted both before and after surface treatment. To ensure consistent evaluation and to minimize the influence of edge effects, three measurement areas of 4 × 4 mm were selected on each sample (Figure 3b).

Surface morphology characterization was further performed employing a scanning electron microscope Phenom ProX (Phenom-World BV, Eindhoven, The Netherlands) (see Table 6 for technical specifications). The imaging was carried out at an accelerating voltage of 15 kV. The beam current was controlled automatically by the Phenom ProX system using its standard high-resolution imaging mode. The instrument facilitated high-resolution imaging, thereby allowing detailed examination of treatment-induced alterations on the surface of the Ti-6Al-4V titanium alloy.

## 3. Results and Discussion

### 3.1. Analysis of Surface Topography After Dry Ice Blasting Method

The mechanisms of dry ice and laser cleaning are governed by distinct yet complementary physical effects. In the dry ice cleaning process, contaminant removal is primarily driven by the combined mechanical impact and thermal shock induced by CO_2_ particles. Upon impact, the rapid sublimation of CO_2_ leads to local cooling and the formation of micro-explosions that detach surface contaminants without damaging the substrate. Increasing the working air pressure and CO_2_ mass flow rate enhances the kinetic energy and momentum of particles, resulting in more efficient removal of loosely bound surface oxides and contaminants. Under these conditions, the treated surfaces exhibit improved uniformity and reduced residual contamination, which confirms the strong correlation between process parameters and cleaning efficiency.

In laser cleaning, a shorter focal length reduces the laser spot size and increases the energy density on the surface, intensifying ablation and localized melting of surface contaminants. Higher fluence at shorter focal lengths promotes the formation of micro-craters and localized melting, while longer focal lengths result in smoother surfaces due to lower energy concentration. This highlights how laser process parameters—particularly focal length and energy density—influence the extent of contaminant removal and surface morphology.

This is further supported by the SEM observations, where surface morphology changes directly reflect the intensity and nature of the applied cleaning mechanisms.

**Figure 4:**
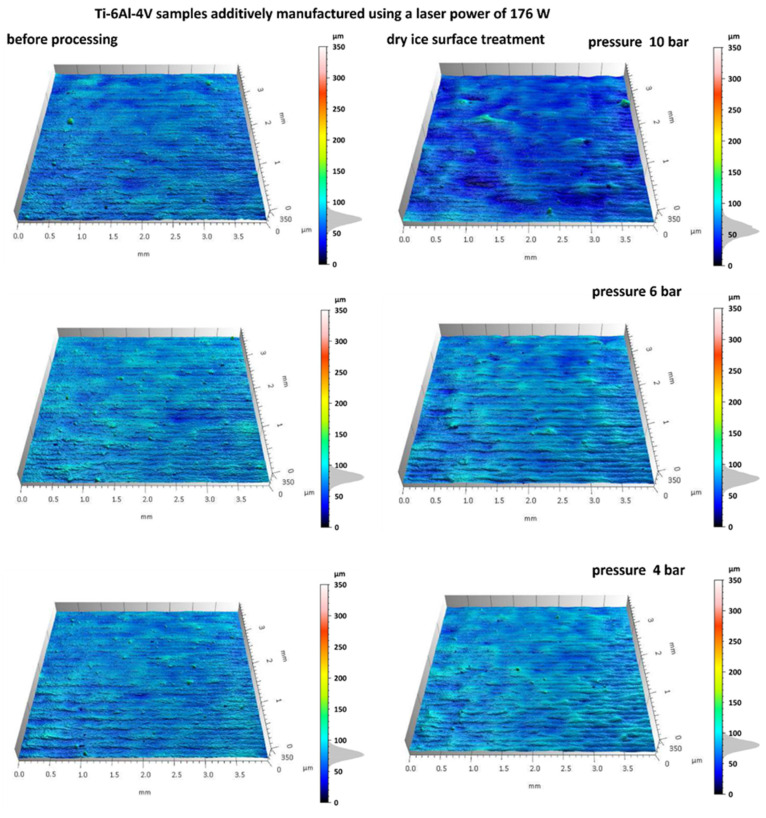
Samples additively manufactured by SLM with S_176 strategy before and after dry ice blasting.

**Figure 5:**
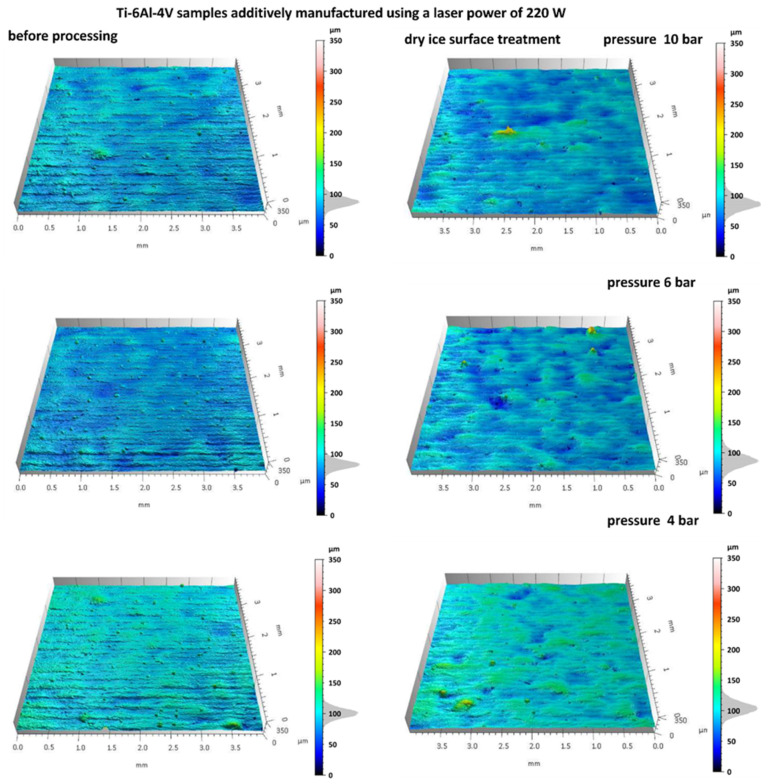
Samples additively manufactured by SLM with S_220 strategy before and after dry ice blasting.

Figure 4 and Figure 5 shows the surface topography of Ti-6Al-4V samples fabricated via SLM before and after dry ice blasting. Before treatment, the surfaces of all samples are clearly irregular, including distinct scan track marks characteristic of the SLM process and numerous adhered spherical powder particles. Localized micro-pits resulting from uneven material melting are also present.

After dry ice blasting, the sample surfaces appear visually cleaner, with most loose powder particles and contaminants removed. The most significant effects were observed at a pressure of 10 bar, where the majority of adhered spherical particles were eliminated. At lower pressures (6 and 4 bar), the treatment effect is less intense. Nevertheless, a slight reduction in defects, mild smoothing of microstructures, and partial removal of loose powder particles were noted. Increasing the blasting pressure enhances the mechanical interactions between CO_2_ particles and the surface, resulting in more efficient removal of residual powder and improved uniformity of the surface microstructure. A similar non-contact mechanism has been observed in hydrojet surface treatment of Ti-6Al-4V components, which also enables effective contaminant removal while preserving the microstructure [4].

**Figure 6:**
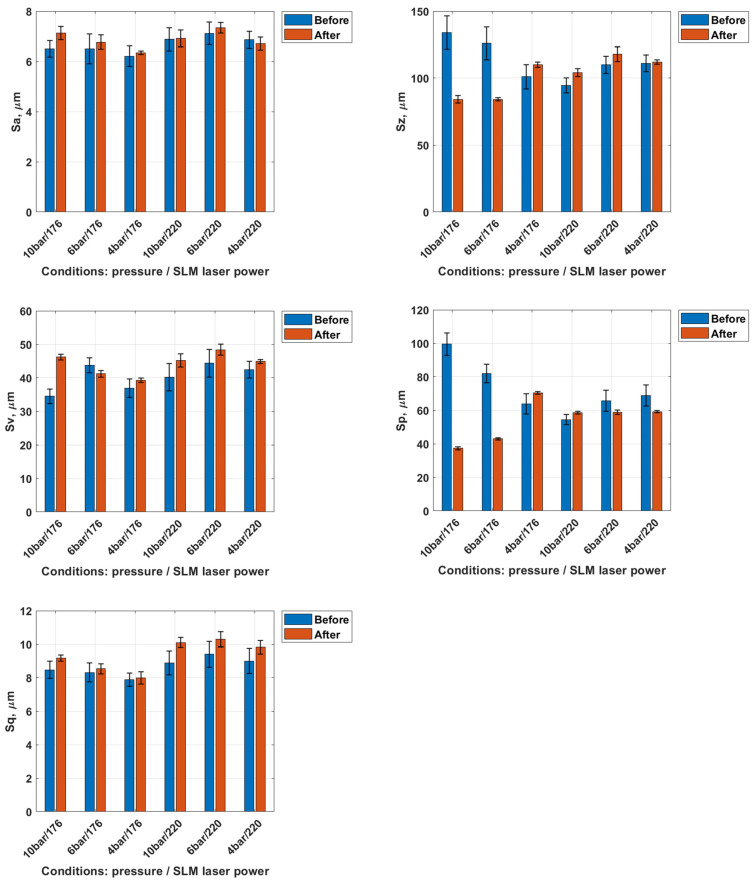
Geometrical surface structure parameters before and after dry ice blasting method.

Subsequently, the surface roughness parameters of the tested samples were analyzed before and after dry ice blasting. The average standard deviation for the measured surface parameters did not exceed ±10%, confirming the good repeatability and reliability of the obtained data. A graphical representation of the results is shown in Figure 6.

The Sa parameter, representing the arithmetic mean height of surface irregularities, exhibited a slight increase for all samples following dry ice treatment. This increase ranged from approximately 5–10%, depending on the process conditions, indicating that the surface became marginally rougher after treatment. This phenomenon may result from the partial removal of loose material, which exposes finer surface features. For example, in the sample treated at 10 bar with 176 W laser power, Sa increased from 6.50 ± 0.33 µm to 7.13 ± 0.14 µm. However, these changes are minor and do not significantly affect the overall level of micro-roughness.

The Sz parameter, representing the maximum profile height, also increased in most cases following dry ice treatment. A decrease in this parameter was observed only for the first two samples (10 bar/176 and 6 bar/176). An increase in Sz reflects a larger height difference between the surface peaks and valleys. This effect may result from the localized removal of material, exposing deeper features of the layer, or from variations in surface topography induced by the cleaning process. In the same sample, Sz decreased from 134 ± 11.1 µm to 84.2 ± 3.5 µm.

The Sv parameter (valley depth) increased on average by 5–15%, while the Sp parameter (peak height) decreased by approximately 50–60% in two cases at an SLM laser power of 176 W and pressures of 10 and 6 bar. The increase in Sv indicates deepening of the valleys, whereas the pronounced decrease in Sp reflects the effective removal of surface peaks. In the representative 10 bar/176 W configuration, Sv increased from 34.5 ± 3.3 µm to 46.2 ± 1.7 µm, while Sp dropped significantly from 99.5 ± 8.0 µm to 37.4 ± 1.5 µm. For the Sq parameter, representing the root mean square deviation of surface heights, the increase after treatment ranged from 5 to 15%. In the same case, Sq increased from 8.47 ± 0.49 µm to 9.17 ± 0.29 µm.

These changes in geometrical surface structure parameters suggest that the dry ice blasting process acts selectively, removing material from protrusions while simultaneously exposing deeper regions of the microstructure. The results demonstrate a distinct change in surface profile characteristics, particularly in peak suppression and valley deepening, as a function of treatment parameters. As a result, the surface after treatment appears cleaner, with reduced surface contamination and partially removed protrusions.

Dry ice blasting results in a slight reduction in the surface roughness of Ti-6Al-4V samples as also observed by Amon et al. [27], who demonstrated that increasing blasting pressure improves cleaning efficiency while preserving surface integrity. The most pronounced smoothing effect was observed for the sample treated at a dry ice pressure of 10 bar, which was fabricated via SLM using a laser power of 220 W. This indicates that local contaminants (such as burn marks) and loose spherical powder particles were effectively removed.

### 3.2. Analysis of Surface Topography After Laser Treatment

**Figure 7:**
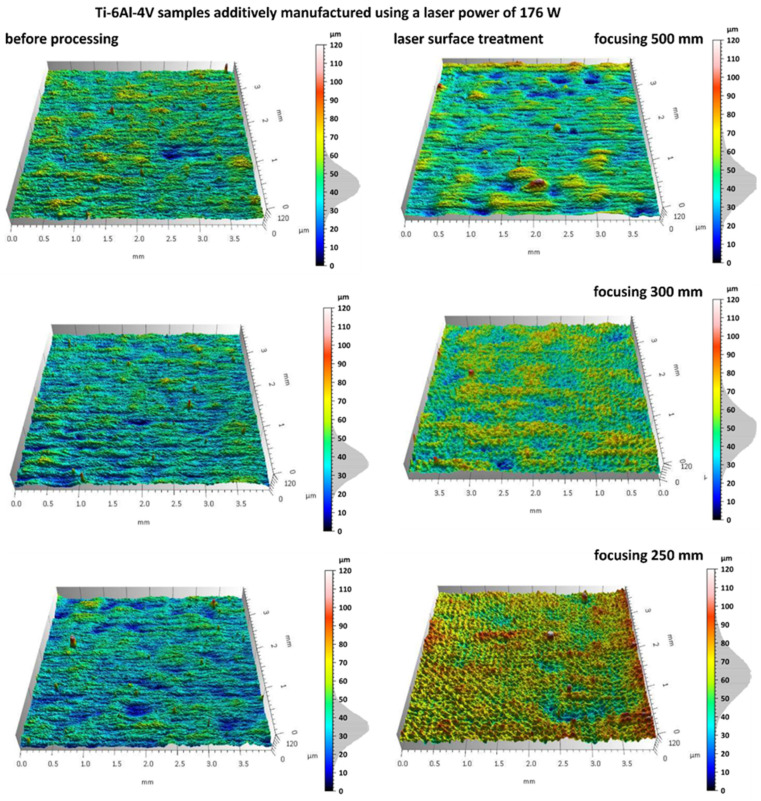
Samples additively manufactured by SLM with S_176 strategy before and after laser treatment.

**Figure 8:**
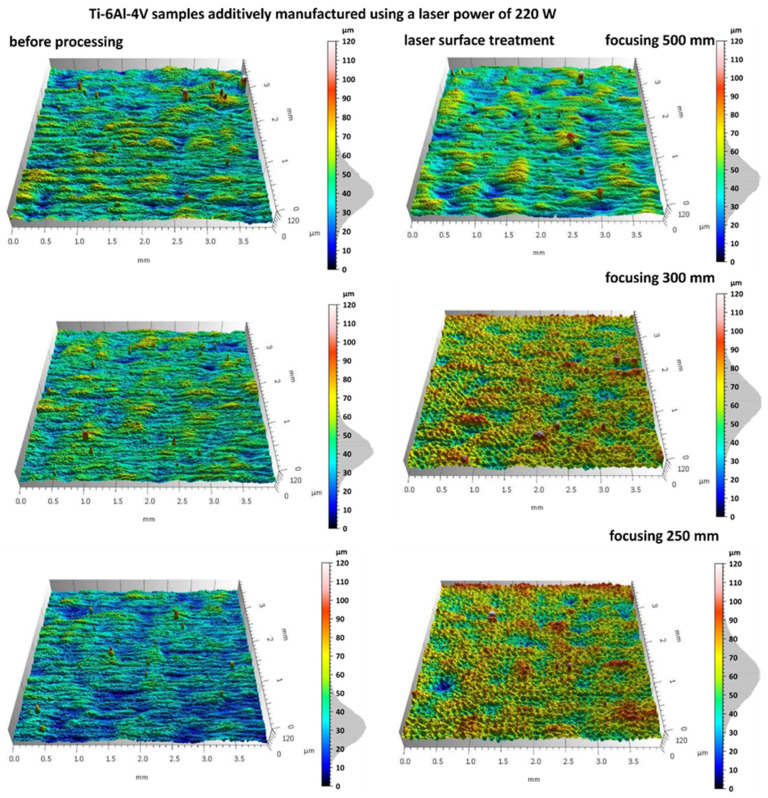
Samples additively manufactured by SLM with S_220 strategy before and after laser treatment.

Additively manufactured Ti-6Al-4V titanium alloy specimens were subjected to laser surface treatment using a 100 W laser system with adjustable focal length. The processing was performed at varying focal distances (500 mm, 300 mm, and 250 mm) in order to assess their influence on the efficiency of the surface treatment. Figure 7 and Figure 8 present images of selected sample surfaces produced by the SLM method (at 176 W and 220 W) before and after laser surface treatment.

Before laser treatment, the surfaces exhibited significant porosity along with irregular features originating from the additive manufacturing process and the overlapping of successive powder layers during layer-by-layer deposition.

Laser treatment with a beam focal length of 500 mm, a distinct remelting of the surface layer was observed, as previously shown in studies on laser polishing of Ti-6Al-4V [8,12], where energy density and focal length were identified as key parameters for optimizing surface quality and minimizing thermal damage. This process resulted in a reduction in micro-irregularities and partial elimination of traces from the additive manufacturing process, suggesting that under these conditions the laser energy was sufficient to achieve controlled melting of a thin material layer without inducing excessive thermal effects.

In the case of laser processing performed with shorter focal lengths of 300 mm and 250 mm, a more irregular surface structure was observed. Under these conditions, the higher energy density resulted from a smaller laser spot diameter at the surface, leading to a greater concentration of energy per unit area. Consequently, more intense melting and vaporization of the material occurred, which promoted the formation of micro-craters, localized remelting zones, and an overall increase in surface roughness amplitude.

Similar phenomena were observed in the results presented in Figure 7, which show the surfaces of samples produced by the SLM method using a laser power of 220 W, both before and after laser surface cleaning with beams of varying focal lengths. In this case as well, a beneficial surface-smoothing effect was observed after laser treatment at a focal length of 500 mm, whereas shorter focal lengths (300 mm and 250 mm) resulted in increased surface irregularity.

The results of microscopic analyses presented in Figure 7 and Figure 8 confirm that the focal length of the laser beam has a significant influence on the surface morphology after treatment. This is consistent with findings from Li et al. [12] and Valentinčič et al. [20], who emphasized the importance of focal distance in controlling microstructural effects and crack formation. A greater focal distance (500 mm) promotes the formation of a more homogeneous surface, whereas a reduction in focal length leads to decreased microstructural uniformity due to excessive thermal effects. Therefore, appropriate selection of the optical and energy parameters of the process is crucial for optimizing the surface quality of additively manufactured titanium alloy components.

**Figure 9:**
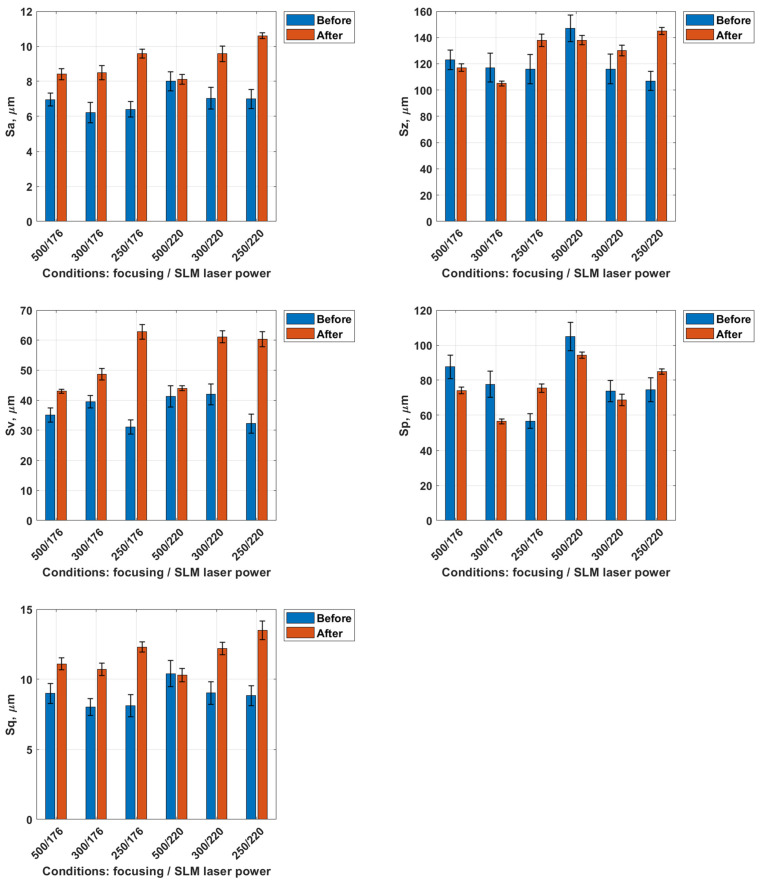
Geometrical surface structure parameters before and after laser.

In the subsequent stage, the surface geometric structure parameters after laser treatment were analyzed. The average standard deviation for the measured surface parameters did not exceed ±10%, confirming the good repeatability and reliability of the obtained data. Figure 9 presents a summary of the results of the topographical parameter analysis (Sa, Sz, Sv, Sp, Sq) for titanium samples produced by the SLM method using laser powers of 176 W and 220 W, both before and after laser surface treatment performed with different focal distances (500 mm, 300 mm, and 250 mm).

The Sa parameter values for example, in the sample treated at a focal length of 500 mm, Sa increased from 6.96 ± 0.35 µm to 8.41 ± 0.28 µm, corresponding to a rise of about 20%. In contrast, at 250 mm, the increase reached 50–60%, with Sa values exceeding 9.5 µm. A similar trend was observed for samples fabricated with S_220 strategy, where laser cleaning at focal lengths of 300 mm and 250 mm resulted in an increase in Sa parameter from around 6.5 µm to approximately 9.5–10.5 µm (a rise of 45–60%). The smallest increase in Sa parameter was again recorded after laser cleaning with a 500 mm focal length, confirming the beneficial effect of lower energy density on surface smoothing.

The Sz parameter values in the 500 mm/176 W case, Sz decreased slightly from 123 ± 9.1 µm to 117 ± 4.7 µm (~5%), indicating modest surface leveling. At 250 mm, Sz increased significantly (e.g., from 116 µm to 138–145 µm), consistent with local melting and the formation of topographic protrusions and pits. After laser treatment, the largest increases in Sz parameter (15–40%) were observed for focal lengths of 300 mm and 250 mm, where local remelting led to an increase in the amplitude of surface irregularities. In contrast, for the 500 mm focal length, a slight decrease in value of Sz parameter (approximately 5–10%) was recorded.

For the Sv parameter, related to valley depth, laser treatment caused a pronounced increase in most cases. For the 500 mm/176 W sample, Sv grew from 35.1 ± 2.7 µm to 43.0 ± 2.2 µm, while at 250 mm, values rose above 60 µm, nearly doubling in some samples. This confirms that short focal lengths tend to expose and deepen subsurface valleys due to local remelting and recoil effects.

For the 500 mm focal length, the value of Sp parameter decreased from approximately 90 µm to 75 µm (about −15%) for samples produced with S_176 strategy. A similar decrease of around 10% was observed for samples fabricated with S_220 strategy. The reduction in Sp indicates flattening of the highest surface peaks due to remelting of the thin top layer. In contrast, at the shortest focal length (250 mm), Sp increased by approximately 10–20%, confirming a deterioration in the uniformity of the surface topography.

The value of Sq parameter increased in most cases by approximately 20–30% after laser treatment. The lowest Sq values were obtained for the 500 mm focal length, regardless of manufacturing strategy, confirming that a greater focal distance promotes the formation of a surface with a more homogeneous microstructure.

**Figure 10:**
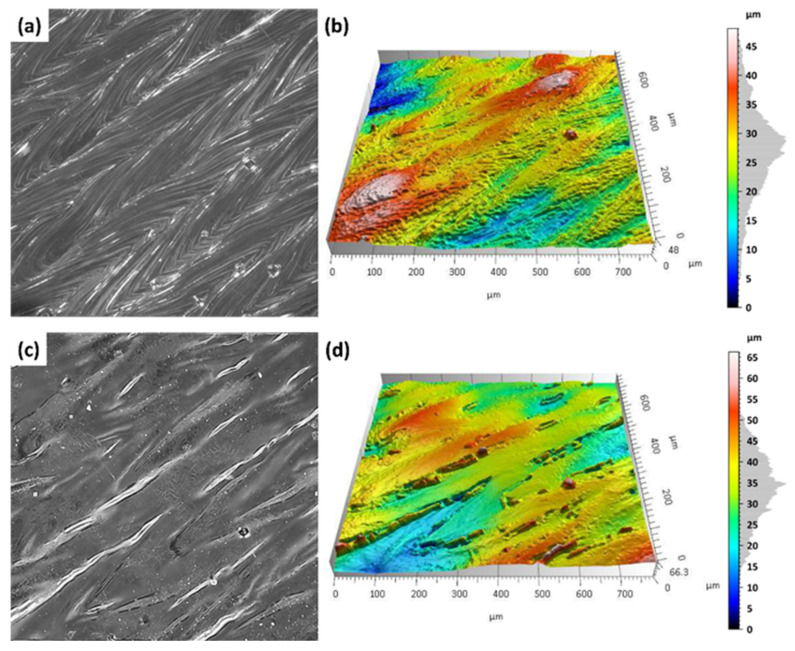
Surface morphology and topography of Ti-6Al-4V samples fabricated using the S_176 SLM strategy: (**a**,**b**) prior to laser treatment; (**c**,**d**) following laser treatment.

On the additively manufactured samples, the Marangoni effect (Figure 10a,b). The molten metal moves within the melt pool, and its flow is driven by the temperature dependence of the surface tension and the density of the alloy [32]. Figure 10 presents representative samples produced by the SLM with S_176 strategy before and after laser treatment with a focal length of 500 mm.

Figure 10a,b show the surface condition before laser treatment, where clear laser paths characteristic of the SLM process are visible. Characteristic laser scan paths from the SLM process are clearly visible, accompanied by an irregular distribution of material. This heterogeneous surface morphology, with local peaks and depressions, is the result of the Marangoni effect—a fluid dynamic phenomenon driven by temperature-induced variations in surface tension during laser melting—as described by Yang et al. [32].

After laser surface treatment with a focal length of 500 mm (Figure 10c,d), a significant improvement in surface homogeneity was observed. This aligns with prior observations [20], which demonstrated that optimized laser parameters can eliminate surface heterogeneity and improve uniformity without inducing microcracks. Height differences in the microstructures were reduced, and the Marangoni effect was eliminated, leading to a leveling of the top layer. No microcracks were detected, confirming that the applied process parameters provide a mild, controlled thermal impact.

**Figure 11:**
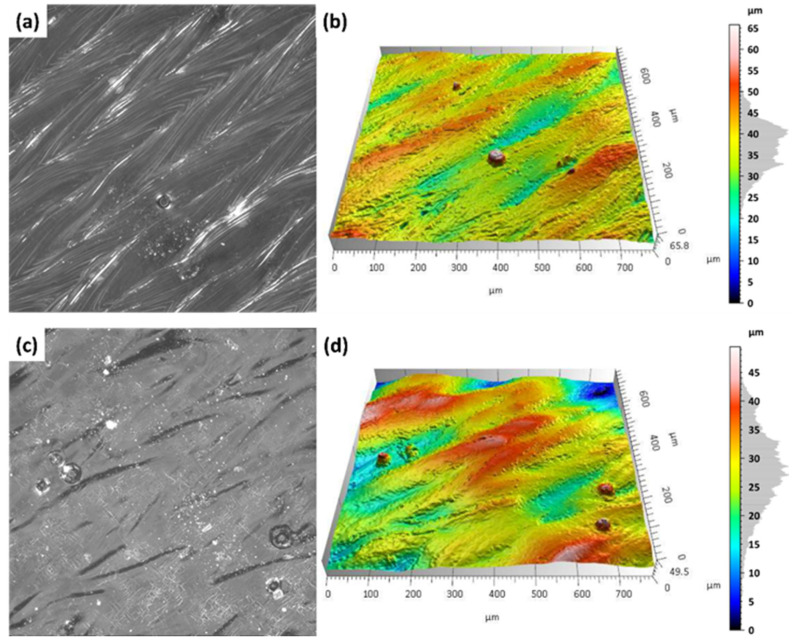
Surface morphology and topography of Ti-6Al-4V samples fabricated using the S_220 SLM strategy: (**a**,**b**) prior to laser treatment; (**c**,**d**) following laser treatment.

Figure 11a,b illustrate the surface condition prior to laser treatment, where, similarly to the previously analyzed samples, laser paths are clearly visible. The surface exhibits a heterogeneous structure with numerous micro-peaks and valleys, as well as the presence of isolated spherical powder particles.

After laser surface treatment at a focal length of 500 mm (Figure 11c,d), the Marangoni effect was eliminated, and a clear smoothing and homogenization of the surface were observed. The top layer structure became more regular, without any signs of microcracks or excessive remelting, indicating a mild and controlled thermal impact of the laser beam. For comparison, at shorter focal lengths (300 mm and 250 mm), a more irregular surface morphology was observed, along with the formation of microcracks resulting from more intense thermal effects and localized remelting of the material.

### 3.3. Analysis of Surface Morphology

**Figure 12:**
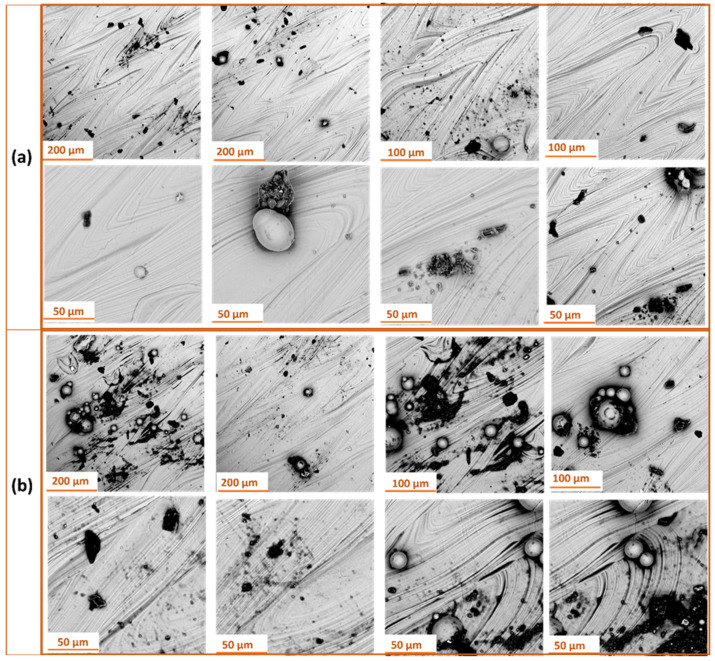
SEM micrographs of Ti-6Al-4V samples fabricated via SLM using: (**a**) the S_176 strategy; (**b**) the S_220 strategy.

Initially, SEM analysis was conducted on additively manufactured samples produced by the SLM method with S_176 and S_220 strategy (Figure 12). The results and are consistent with previous observations on as-built surface morphology of SLM Ti-6Al-4V samples reported by Pal et al. [33].

Analysis of the obtained SEM images revealed that the surface of samples produced with S_176 strategy is more homogeneous, with a small number of fine powder particles and regularly distributed melting traces. Increasing the laser power to 220 W resulted in a significant rise in surface contaminants in the form of spherical splatters, adhered particles, as well as localized remelting and micropores. Higher laser power during sample fabrication promotes the formation of defects and contaminants, which may negatively affect the functional properties of the manufactured components.

**Figure 13:**
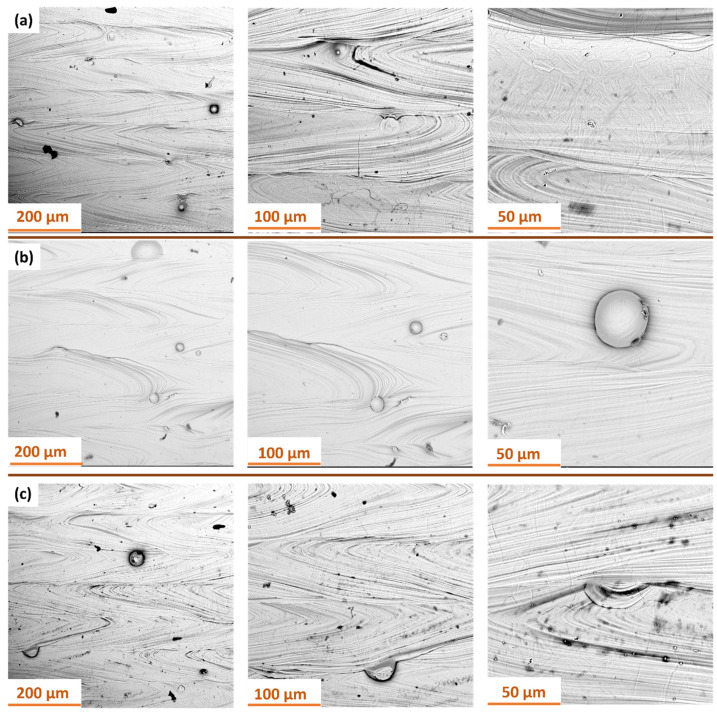
SEM images of Ti-6Al-4V samples produced with S_176 strategy after dry ice blasting at different pressure and mass flow rate: (**a**) 10 bar and 50 kg/h; (**b**) 6 bar and 40 kg/h; (**c**) 4 bar and 30 kg/h.

Figure 13 presents SEM images of the surface of samples manufactured with S_176 strategy after dry ice blasting method carried out with different process parameters. All images show characteristic traces of directional layer melting formed during the additive manufacturing process. At the highest pressure of 10 bar (Figure 13a), the surface after dry ice cleaning is the cleanest, with most particles removed. Small localized indentations in the material are still visible.

At 6 bar (Figure 13b), the treatment primarily removes minor surface contaminants, without noticeable alteration of the surface structure. The layer topography remains intact. At 4 bar (Figure 13c), the cleaning effectiveness is insufficient—contaminants formed during the additive process remain, indicating inadequate kinetic energy.

**Figure 14:**
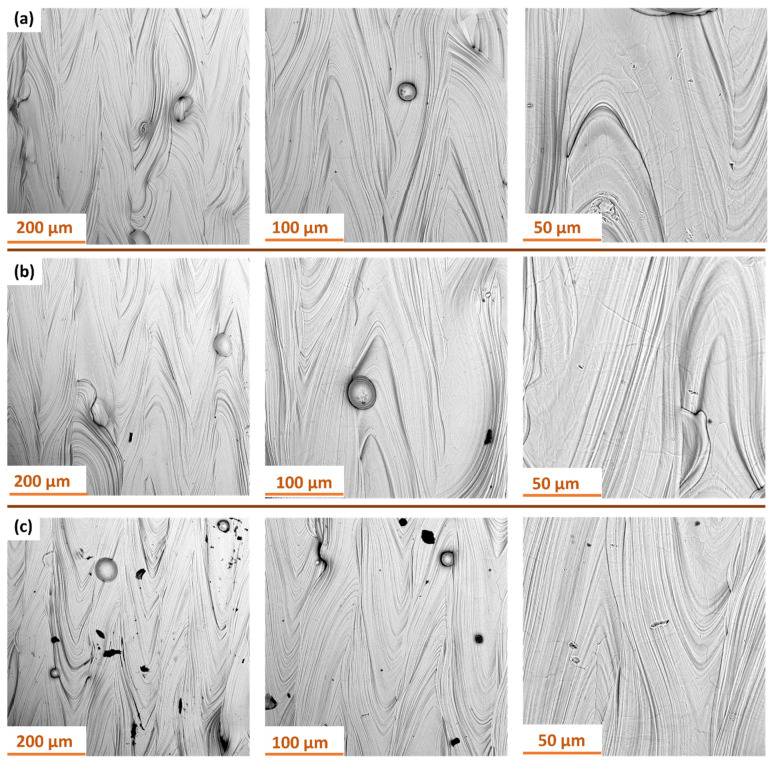
SEM images of Ti-6Al-4V samples produced with S_220 strategy after dry ice blasting at different pressure and mass flow rate: (**a**) 10 bar and 50 kg/h; (**b**) 6 bar and 40 kg/h; (**c**) 4 bar and 30 kg/h.

Figure 14 presents SEM images of the surface of samples manufactured with S_220 strategy after dry ice blasting method carried out with different process parameters. All samples show characteristic, parallel traces of the layers created during the additive manufacturing process. The surface structure of the material remains intact regardless of the processing parameters used. Dry ice blasting did not cause any melting or geometric deformation of the layers. The only noticeable effect of CO_2_ particle interaction with the surface are small, local scratches, indicating minimal mechanical interference with the material. At the highest process parameters (Figure 14a), the surface is cleanest—most of the contaminants in the form of burnt particles have been removed. At lower pressures (Figure 14b), a few powder residues are present. However, at the lowest pressure and flow (Figure 14c), numerous contaminants and particles are still visible on the surface, indicating low cleaning efficiency under these conditions. These findings confirm that contaminant removal during dry ice blasting is governed by both kinetic and thermodynamic effects. Higher air pressure and CO_2_ mass flow rate enhance particle momentum and heat transfer, promoting micro-explosions that detach contaminants without damaging the substrate. Thus, the process efficiency is directly correlated with these parameters, which determine the extent of surface cleaning and the final morphology.

**Figure 15:**
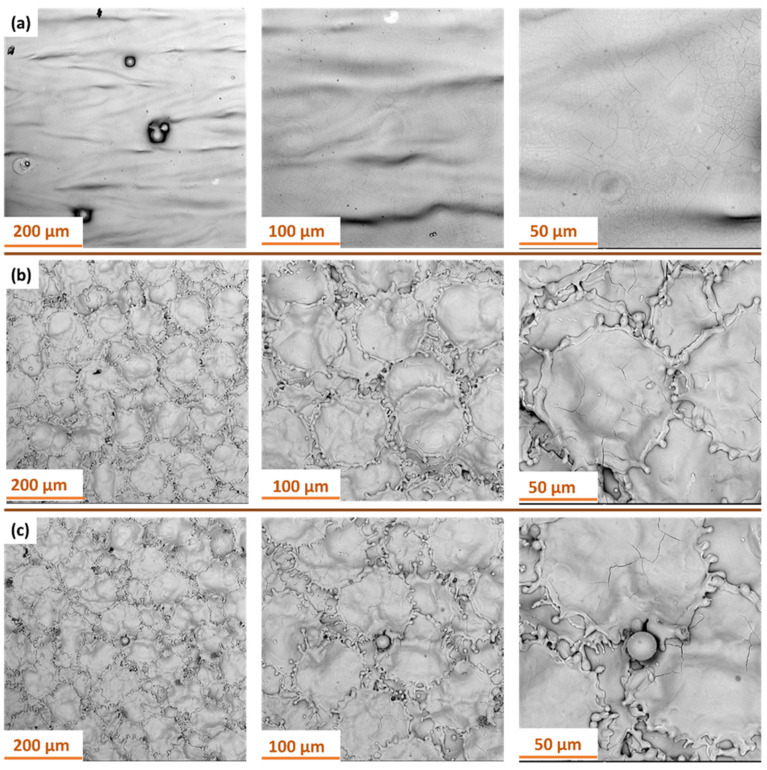
SEM images of Ti-6Al-4V samples produced with S_176 strategy after laser treatment at different focal lengths: (**a**) 500 mm; (**b**) 300 mm; (**c**) 250 mm.

In the next stage, SEM analysis was conducted to evaluate the surface of Ti-6Al-4V titanium alloy produced by additive SLM with S_176 strategy after laser surface treatment at different focal lengths. Figure 15 presents SEM images at various magnifications.

In laser cleaning, the dominant mechanisms are photothermal and photomechanical effects. The absorbed laser energy causes rapid heating, melting, and partial vaporization of contaminants, while the resulting pressure waves assist in their ejection from the surface. The focal length of the laser directly influences the local energy density—shorter focal lengths increase fluence and intensify ablation, whereas longer focal lengths promote gentle surface smoothing.

For the 500 mm focal length (Figure 15a), the surface retains a smooth profile with the presence of localized defects and minor remelted features. As the focal length decreases (Figure 14b,c), a clear intensification of thermal effects is observed, leading to the formation of characteristic, more complex morphological structures.

At a focal length of 300 mm (Figure 15b), the surface exhibits a well-defined, quasi-spherical texture resembling convex islands surrounded by narrow depressions—an effect likely associated with localized remelting and rapid solidification of the material. The boundaries of these structures show microporosity and irregular cracks.

Further reducing the focal length to 250 mm (Figure 15c) results in an even more intricate and pronounced surface texture. The remelted structures form irregular, interconnected networks of protrusions, often accompanied by thermal cracks. Additionally, defects in the form of spherical particles (globules) approximately 20 μm in diameter, which were not removed during the treatment, are noticeable. The observed changes clearly indicate an increase in the intensity of the thermal impact in the laser-affected zone.

**Figure 16:**
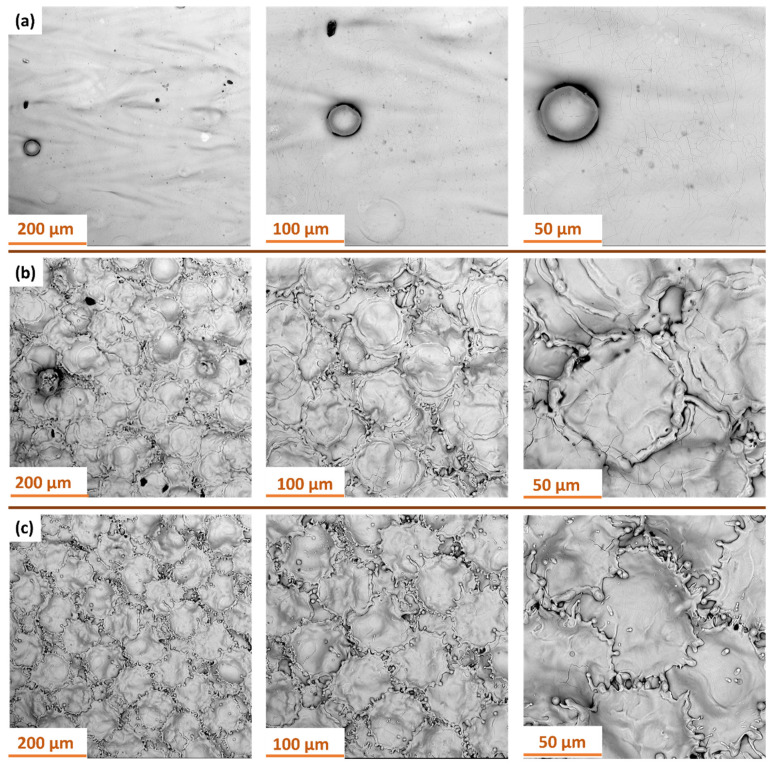
SEM images of Ti-6Al-4V samples produced with S_220 strategy after laser treatment at different focal lengths: (**a**) 500 mm; (**b**) 300 mm; (**c**) 250 mm.

Figure 16 shows SEM images of the surface of Ti-6Al-4V samples produced by SLM with the S_220 strategy, following laser treatment with a 100 W laser at various focal lengths.

For the 500 mm focal length (Figure 16a), the surface after treatment exhibits a relatively smooth morphology with a small number of remelting traces. Individual spherical globules originating from the additive manufacturing process are visible, representing residual unmolten powder that was not removed during laser treatment. The presence of these particles indicates a low intensity of thermal impact at this focal length.

At a focal length of 300 mm (Figure 16b), the surface is covered with an extensive structure resembling irregular, convex islands separated by a network of microporous boundaries. This type of texture results from increased localized remelting and rapid solidification of the material, which promotes the formation of local microcracks.

At a focal length of 250 mm (Figure 16c), a further increase in the intensity of the thermal impact is evident. The surface exhibits a continuous, highly undulated structure with numerous microcracks and pronounced remelting boundaries.

Shortening the focal length reduces the laser spot diameter and increases the local energy density, which intensifies remelting and promotes the formation of morphological irregularities. These findings confirm that both the optical system configuration and process conditions play a crucial role in shaping the relationship between contaminant removal efficiency and surface integrity preservation.

## 4. Conclusions

This study investigated the impact of laser and cryogenic (dry ice blasting) surface treatments on the surface characteristics of Ti-6Al-4V alloy samples fabricated using Selective Laser Melting (SLM). The primary objective was to assess the effectiveness of both techniques in eliminating surface contaminants and enhancing surface structure and roughness. The main conclusions drawn from the results are as follows:

1. Cryogenic dry ice blasting effectively removed residual powder and oxide films from as-built SLM surfaces. For instance, in samples fabricated with the S_176 strategy and treated at 10 bar, the Sp parameter dropped from 99.5 µm to 37.4 µm, indicating significant peak removal. Concurrently, Sv increased from 34.5 µm to 46.2 µm, exposing deeper valleys, while Sz decreased from 134 µm to 84.2 µm, reflecting a reduced overall profile height. Moderate increases in Sa (from 6.5 to 7.13 µm) and Sq (from 8.47 to 9.17 µm) were consistent with selective top-layer removal.
2. The best surface cleaning effects were achieved with dry ice at 10 bar and CO2 flow of 50 kg/h, resulting in surfaces with minimal residual contamination and visibly improved cleanliness.
3. Laser treatment induced surface changes dependent on focal length. At a 500 mm focal length, thermal effects were moderate—Sp decreased by ~15% and Sq increased by only 10–15%, indicating controlled surface smoothing with minimal remelting.
4. In contrast, reducing the focal length to 250 mm led to a 20–40% increase in Sp and Sv, suggesting intensified thermal gradients, local remelting, and potential microcrack formation.
5. Compared to mechanical or chemical cleaning, dry ice blasting offers a non-contact, environmentally friendly solution for titanium alloys, preserving microstructure while effectively removing surface contaminants.
6. Dry ice improves surface cleanliness and uniformity, even if roughness increases slightly. Laser treatment allows targeted morphology control, with its impact tunable by focal length. These results emphasize the importance of process optimization in relation to intended application requirements, especially for aerospace and biomedical components, where surface precision, cleanliness, and integrity are essential.
7. Further research should address the influence of these surface treatments on mechanical and electrochemical performance, including fatigue resistance and corrosion behavior of SLM Ti-6Al-4V, to validate their suitability in demanding operational environments.
8. Overall, cleaning effectiveness is closely tied to physical mechanisms involved: dry ice relies on mechanical and cryogenic interaction, while laser processing is governed by photothermal energy absorption and localized melting dynamics.

## Figures and Tables

**Table 1:** SLM operating parameters.

Parameter	Strategy Symbol	
	S_176	S_220
Laser power, P [W]	176	220
Scanning speed, Ss [mm/s]	880	1100
Hatching distance, Hd [mm]	0.1	
Layer thickness, Lt [mm]	0.03	
Volumetric energy density, E_v_ [J/mm^3^]	67	

**Table 2:** Chemical composition of Ti-6Al-4V titanium alloy powder (wt. %) [4].

	Ti	Al	V	Fe	O	C	N	H
Ti-6Al-4V	Balance	6.00	4.00	≤0.25	≤0.13	≤0.08	≤0.03	≤0.012

**Table 3:** Technical parameters of the Ares 50Z device.

Parameter	Value
CO_2_ mass flow rate, [kg/h]	0–72
CO_2_ granulate diameter, [mm]	10 (max)
Granulate container capacity, [kg]	<20
Working air pressure, [bar]	0.3–0.5
Power supply, [V]	200–240

**Table 4:** Dry ice blasting process parameters.

Process Number	CO_2_ Mass Flow Rate, [kg/h]	Working Air Pressure, [bar]	Working Nozzle Diameter, [mm]
1	50	10	7
2	40	6	
3	30	4	

**Table 5:** Laser process parameters.

Parameter	Value
Laser wavelength, [nm]	1064
Laser focal length, [mm]	250, 300, 500
Diameter of the scanned area, [mm]	50
Laser pulse frequency, [kHz]	100
Pulse energy, [mJ]	1
Power supply, [V]	230

**Table 6:** Technical specifications of the Phenom ProX scanning electron microscope.

Parameters	Values
Optical zoom (light mode)	27×–160×
SEM magnification (electron optics)	From 160× up to 350,000×
Resolution, nm	≤6 SED, ≤8 BSD
Digital image magnification	Up to 12×
Beam accelerating voltage	5 to 20 kV
Maximum sample width	25 mm (optionally 32 mm)
Maximum sample height	35 mm (optionally up to 100 mm)
Electron source lifespan (CeB_6_)	Up to 1500 h

## Data Availability

The original contributions presented in this study are included in the article. Further inquiries can be directed to the corresponding authors.
